# Long non-coding RNA PICSAR decreases adhesion and promotes migration of squamous carcinoma cells by downregulating α2β1 and α5β1 integrin expression

**DOI:** 10.1242/bio.037044

**Published:** 2018-11-15

**Authors:** Minna Piipponen, Jyrki Heino, Veli-Matti Kähäri, Liisa Nissinen

**Affiliations:** 1 Department of Dermatology, University of Turku and Turku University Hospital, FI-20520 Turku, Finland; 2 Western Cancer Center (FICAN West), University of Turku and Turku University Hospital, FI-20520 Turku, Finland; 3 MediCity Research Laboratory, University of Turku, FI-20520 Turku, Finland; 4 Department of Biochemistry, University of Turku, FI-20500 Turku, Finland

**Keywords:** Long non-coding RNA, PICSAR, Integrin, Adhesion, Cutaneous squamous cell carcinoma, Cancer

## Abstract

Long non-coding RNAs (lncRNAs) regulate various cellular processes, and they have emerged as potential biomarkers and therapeutic targets in cancer. We have previously characterized the oncogenic role of lncRNA PICSAR (p38 inhibited cutaneous squamous cell carcinoma associated lincRNA) in cutaneous squamous cell carcinoma (cSCC), the most common metastatic skin cancer. In this study, we show that knockdown of PICSAR in cSCC cells upregulates expression of α2, α5 and β1 integrins, resulting in increased cell adhesion and decreased cell migration on collagen I and fibronectin. In contrast, overexpression of PICSAR in cSCC cells downregulates expression of α2, α5 and β1 integrins on cell surface, resulting in decreased cell adhesion on collagen I and fibronectin and increased cell migration. These results demonstrate a novel mechanism for regulation of the expression of collagen and fibronectin binding integrins by lncRNA PICSAR, leading to altered adhesion and migration of cSCC cells.

This article has an associated First Person interview with the first author of the paper.

## Abstract (precis)

**Summary:** Long non-coding RNA PICSAR decreases adhesion and promotes migration of cutaneous squamous cell carcinoma cells by regulating the expression of collagen and fibronectin binding α2β1 and α5β1 integrins.

## INTRODUCTION

Long non-coding RNAs (lncRNAs) are a diverse and widely uncharacterized group of non-coding transcripts involved in cell regulation (Geisler and Coller, 2013). The single-stranded RNA structure makes lncRNAs versatile regulators of gene expression, as they are able to bind to chromatin and other DNA sequences, RNA and proteins (Rinn and Chang, 2012). Recent studies have elucidated the role of various lncRNAs in several malignancies, such as neurological and cardiovascular diseases (Cipolla et al., 2018), but also in cancer progression, suggesting them as important biomarkers and therapeutic targets (Evans et al., 2016; Schmitz et al., 2016). However, the understanding of mechanistic functions of distinct lncRNAs in growth and invasion of cancer cells is incomplete.

Keratinocyte-derived cutaneous squamous cell carcinoma (cSCC) is the most common metastatic skin cancer, and its incidence is increasing globally (Madan et al., 2010; Ratushny et al., 2012). An important risk factor for cSCC is the exposure to solar ultraviolet light, but the tumor microenvironment plays also a key role in the progression of cSCC (Nissinen et al., 2016). The molecular basis of the progression of cSCC is incompletely understood. We have recently identified and characterized lncRNA PICSAR (p38 inhibited cutaneous squamous cell carcinoma associated lincRNA), which is specifically expressed by tumor cells in cSCC but not by keratinocytes in normal skin *in vivo* (Piipponen et al., 2016). We showed that knockdown of PICSAR inhibits cSCC cell proliferation and migration on an uncoated surface and suppresses growth of human cSCC xenografts *in vivo*, providing evidence for its tumorigenic function (Piipponen et al., 2016).

Here, we show that knockdown of PICSAR in cSCC cells upregulates expression of α2, α5 and β1 integrins in cSCC cells, resulting in increased cell adhesion and decreased cell migration both on collagen I and fibronectin. In contrast, overexpression of PICSAR in cSCC cells downregulates expression of α2, α5 and β1 integrins on cell surface, resulting in decreased cell adhesion on collagen I and fibronectin and increased cell migration. These results identify a novel mechanistic role for long non-coding RNA PICSAR in modifying the expression of collagen- and fibronectin-binding integrins, and cell adhesion and migration.

## RESULTS AND DISCUSSION

### Knockdown of PICSAR inhibits cSCC cell migration on collagen I and fibronectin

**Fig. 1.**
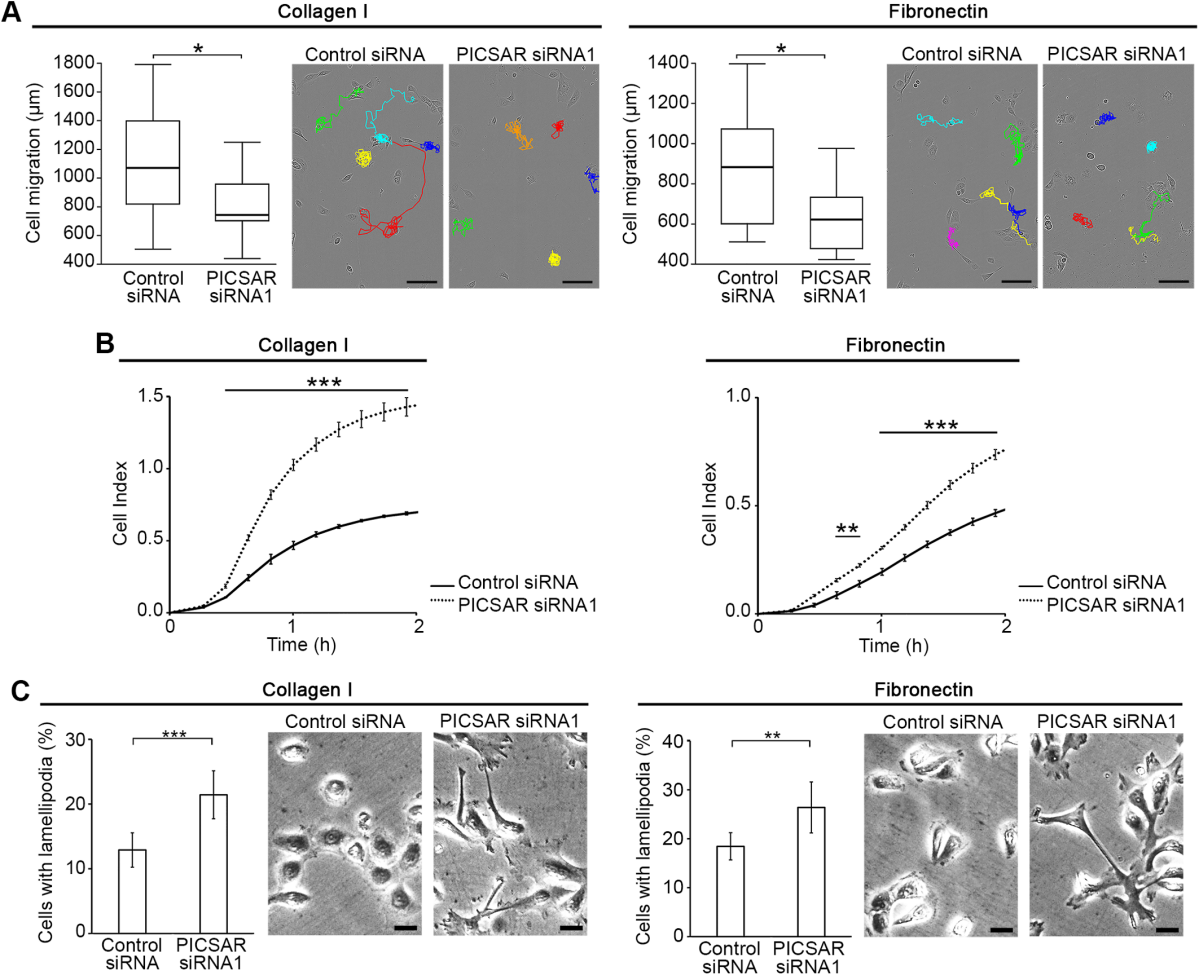
**Knockdown of PICSAR inhibits cSCC cell migration and increases cSCC cell adhesion and formation of lamellipodia.** cSCC cells (UT-SCC12A) were transfected with PICSAR siRNA (siRNA1) or control siRNA. (A) Cells were plated on collagen I or fibronectin 72 h after transfection and migration of individual cells was imaged using the IncuCyte ZOOM^®^ real-time cell imaging system. Cell tracking (*n*=15) was quantitated using ImageJ software. Median±s.d. is shown; *P<0.05, Mann–Whitney two-way *U*-test. Representative images of cell tracking are shown. Scale bars: 100 µm. (B) Cells were plated on collagen I or fibronectin coated 96-well electronic microtiter plate 72 h after transfection and cell adhesion was measured using the xCELLigence system (*n*=3). The cell index indicates the readout of the microelectrode impedance which corresponds to the strength of cell adhesion. Mean±s.d. is shown; **P<0.01, ***P<0.001; two-tailed *t*-test. (C) Cells were plated on collagen I or fibronectin 72 h after transfection and fixed 4 h after plating. The number of lamellipodia containing cells was quantitated from microscopic images with a 20x objective (*n*=3). Representative images of the quantification are shown. Scale bars: 10 µm. Mean±s.d. is shown; **P<0.01, ***P<0.001; two-tailed *t*-test.

We have previously provided the first functional evidence of a tumorigenic lncRNA in cSCC, called PICSAR (p38 inhibited cutaneous squamous cell carcinoma associated lincRNA) (Piipponen et al., 2016). To study the functional role for PICSAR in cell motility, we analyzed real-time cell migration of individual cSCC cells on a collagen I and fibronectin coated surface after PICSAR knockdown. The cell tracking assay showed that silencing of PICSAR decreased migration of cSCC cells on collagen I and fibronectin compared to control siRNA transfected cells (Fig. 1A; Fig. S1A).

### Knockdown of PICSAR increases cSCC cell adhesion and formation of lamellipodia

To further examine the role of PICSAR in cell adhesion, cSCC cells were transfected with PICSAR siRNA or control siRNA and plated on collagen I or fibronectin. Real-time cell adhesion was determined using the xCELLigence system. The results showed that PICSAR knockdown significantly increased adhesion of cSCC cells to collagen I and fibronectin compared to control siRNA transfected cells (Fig. 1B; Figs S1B and S2A). After PICSAR knockdown the morphology of cSCC cells was less spherical than in the control siRNA transfected cultures and cells extended multiple lamellipodia around them (Fig. 1C; Figs S1C and S2B). The relative number of cSCC cells with lamellipodia on collagen I and fibronectin was higher in cultures after PICSAR knockdown, as compared to control siRNA transfected cultures (Fig. 1C; Figs S1C and S2B).

### Knockdown of PICSAR increases integrin expression in cSCC cells

**Fig. 2.**
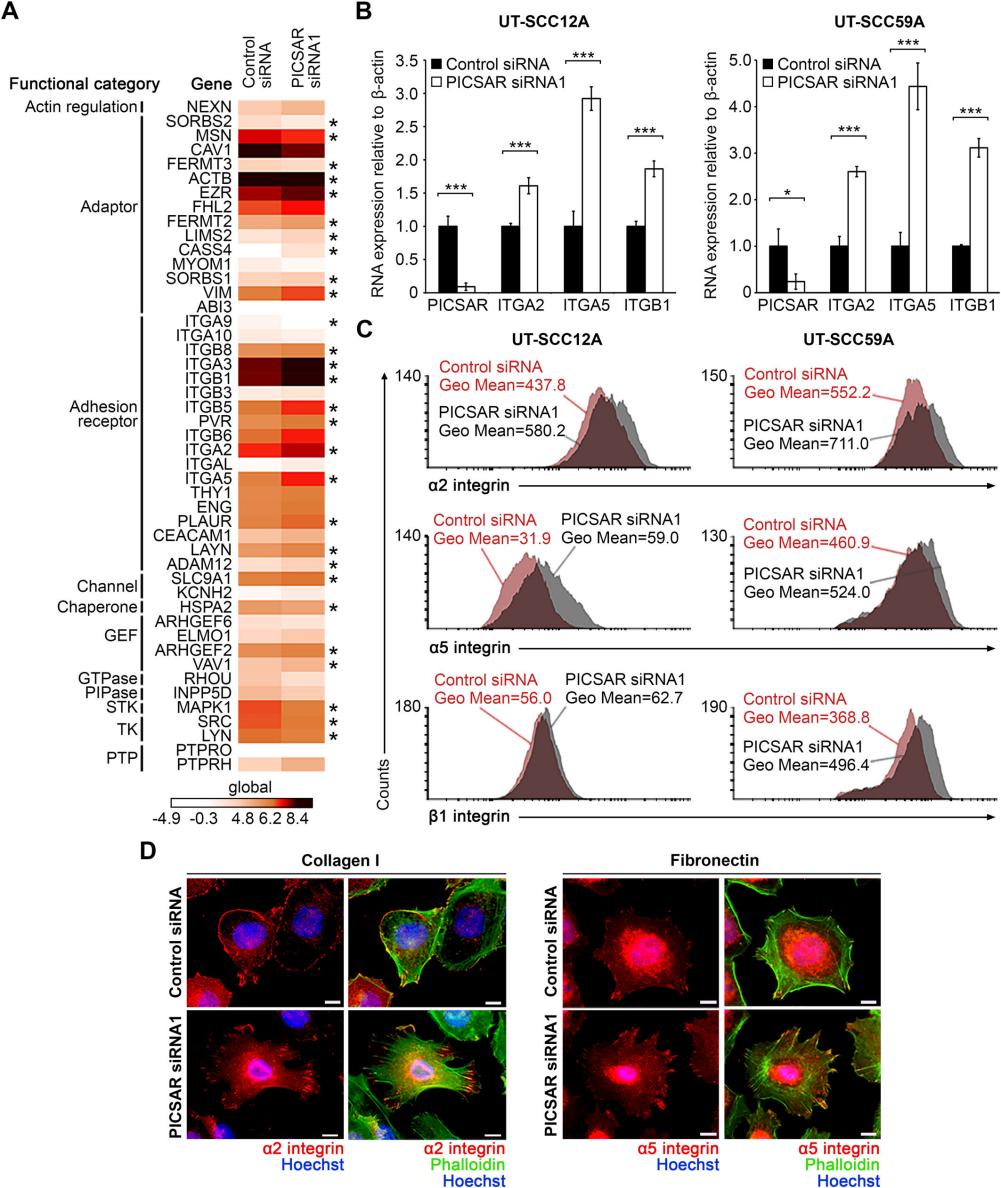
**Knockdown of PICSAR increases integrin expression in cSCC cells.** (A) Three cSCC cell lines (UT-SCC12A, UT-SCC59A, and UT-SCC118) were transfected with PICSAR siRNA (siRNA1) or control siRNA. After 72 h of transfection, whole transcriptome analysis was performed with RNA-seq. Heatmap showing several cell adhesion related genes regulated by PICSAR knockdown (FC log 2>0.5). GEF, guanine nucleotide exchange factor; PIPase, phosphatidylinositol phosphatase; STK, serine/threonine specific protein kinase; TK, tyrosine kinase; PTP, protein tyrosine phosphatase; *P<0.05. (B) Levels of PICSAR and α2, α5 and β1 integrin mRNAs were determined with qPCR in PICSAR knockdown UT-SCC12A and UT-SCC59A cells (*n*=3). Mean±s.d. is shown. *P<0.05, ***P<0.001; two-tailed *t*-test. (C) Flow cytometry was performed to measure α2, α5 and β1 integrin expression on the cell surface in PICSAR knockdown UT-SCC12A and UT-SCC59A cells. (D) UT-SCC12A cells were transfected with PICSAR siRNA or control siRNA and plated on collagen I or fibronectin. Cells were fixed and stained with primary antibodies against α2 or α5 integrins (red), Alexa Fluor^®^ 488 conjugated phalloidin (green) and Hoechst (blue). Scale bars: 10 µm.

To elucidate the mechanistic role of PICSAR in regulating cell adhesion, the differentially expressed genes specifically functioning in cell adhesion (Winograd-Katz et al., 2014) were further examined from the RNA sequencing of cSCC cells after PICSAR knockdown (Piipponen et al., 2016). We noted that the expression of several of these genes was regulated (FC log2>0.5) in cSCC cells after PICSAR knockdown, including genes coding for integrins, particularly *ITGA2*, *ITGA5* and *ITGB1*, which code for α2, α5, and β1 integrin subunits (Fig. 2A). These integrin subunits form functional heterodimeric α2β1 and α5β1 membrane proteins, which function as cell surface receptors for type I collagen and fibronectin, respectively. These results support our previous bioinformatic analysis of PICSAR knockdown RNA sequencing, which revealed extracellular matrix binding and extracellular matrix-receptor interaction among the top functions associated with differentially expressed genes after PICSAR knockdown (Piipponen et al., 2016).

Integrins serve as important regulators of cell migration, differentiation and survival, and they also play a role in cancer progression (Guo and Giancotti, 2004; Heino and Käpylä, 2009). For instance, α2β1 integrin has been shown to exert a tumor suppressive function in breast and prostate cancer, whereas loss of α2β1 integrin correlates with metastasis in cancer patients and promotes tumor cell intravasation *in vivo* and *in vitro* (Ramirez et al., 2011), indicating that loss of integrin-mediated cell adhesion is an important event in invasion and metastasis of cancer cells. Cell migration is a multistep process, which requires focal adhesion disassembly regulated by integrin recycling, and complex coordination of actin cytoskeleton, microtubules and a large group of signaling molecules (Webb et al., 2002; Pellinen and Ivaska, 2006). It is also dependent on the optimal balance in integrin expression, so that increased integrin expression results in increased adhesiveness, as the cells are able to form more bonds to the surrounding extracellular matrix (Palecek et al., 1997).

Quantitation of integrin mRNA levels in cSCC cells after PICSAR knockdown with qPCR showed elevated expression of α2, α5 and β1 integrins in cSCC cells after PICSAR knockdown (Fig. 2B; Fig. S3A). Furthermore, flow cytometry analysis showed increased expression of α2 and α5 integrins on the surface of cSCC cells after PICSAR knockdown, compared to the control siRNA transfected cells (Fig. 2C). Expression of β1 integrin on the cell surface was increased in UT-SCC59A when using two different PICSAR targeting siRNAs (Fig. 2C; Fig. S3B), whereas in UT-SCC12A cells the effect was less potent after PICSAR knockdown (Fig. 2C). Immunofluorescence staining of α2 and α5 integrins showed similar localization to the cell surface and adhesion sites both in control siRNA and PICSAR siRNA transfected cSCC cells (Fig. 2D).

### PICSAR overexpression decreases integrin expression in cSCC cells

**Fig. 3.**
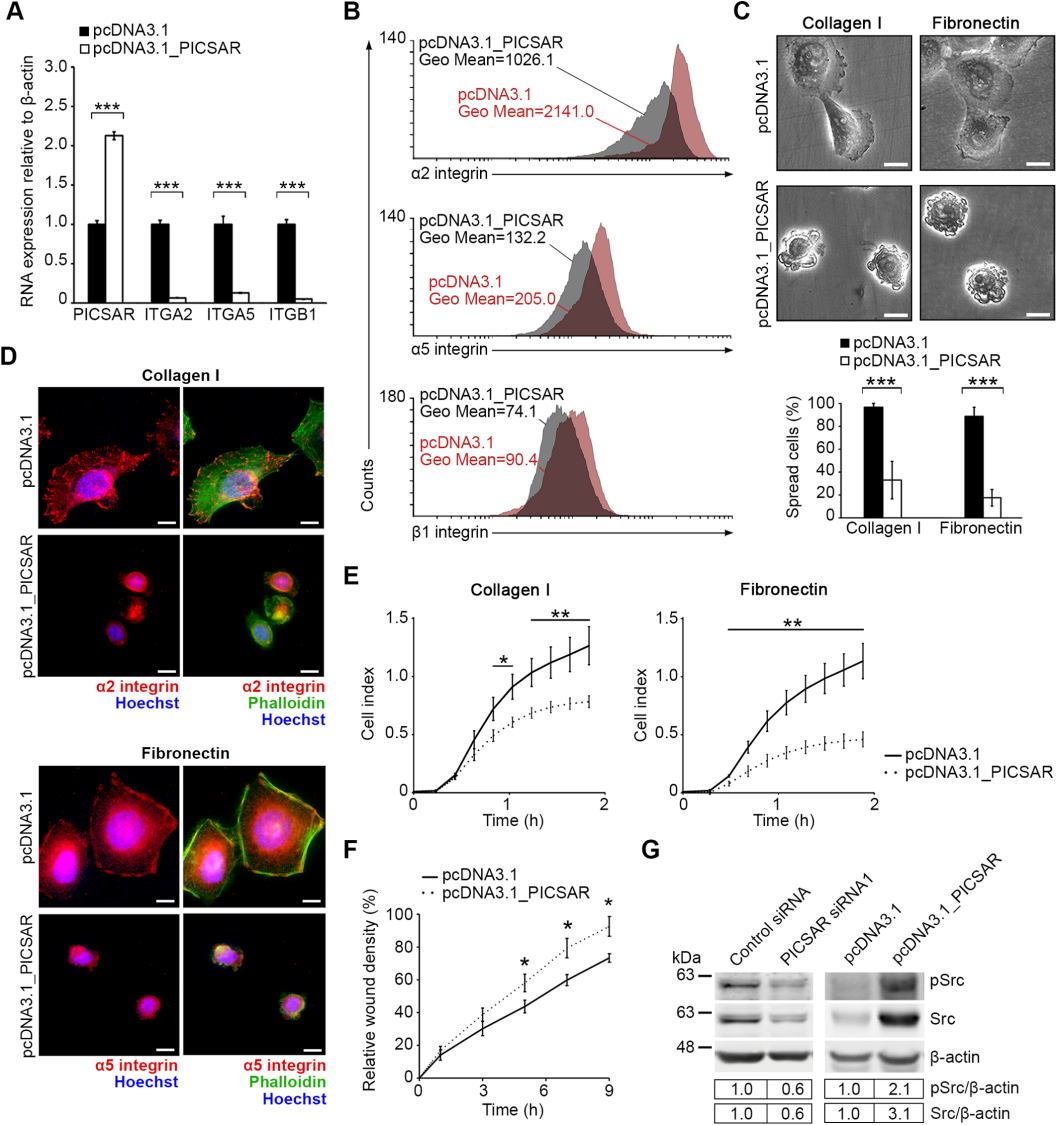
**PICSAR overexpression decreases cell adhesion and spreading, and increases migration of cSCC cells by regulating integrin expression.** UT-SCC59A cells were transfected with PICSAR expression construct (pcDNA3.1_PICSAR) or empty vector (pcDNA3.1) and selective pressure of cell pools was maintained by Geneticin. (A) Expression of PICSAR and α2, α5 and β1 integrin mRNAs was measured using qPCR (*n*=3). Mean±s.d. is shown. (B) Flow cytometry was used to measure α2, α5 and β1 integrin expression on the cell surface. (C) PICSAR overexpressing and control UT-SCC59A cells were plated on collagen I and fibronectin and the number of spread cells was quantitated from microscopic images with a 20x objective (*n*=3). Scale bars: 10 µm. Mean±s.d. is shown. Representative images of the quantification are shown. (D) PICSAR overexpressing and control UT-SCC59A cells were plated on collagen I or fibronectin and stained with primary antibodies against α2 or α5 integrin (red), Alexa Fluor^®^ 488 conjugated phalloidin (green) and Hoechst (blue). Scale bars: 10 µm. (E) Adhesion of PICSAR overexpressing and control UT-SCC59A cells on collagen I and fibronectin was measured using the xCELLigence system (*n*=3). Mean±s.d. is shown. (F) PICSAR overexpressing and control UT-SCC59A cells were plated on a 96-well plate and incubated for 6 h with 1 mM hydroxyurea to prevent cell proliferation. Cell monolayer was scratched and cells were imaged using the IncuCyte ZOOM^®^ real-time cell imaging system (*n*=5). Mean±s.e.m. is shown. (G) Protein levels for phosphorylated and total Src were determined by western blotting analysis of PICSAR siRNA (siRNA1) transfected and stably PICSAR overexpressing UT-SCC59A cells. The representative image and quantification shown are from one individual experiment. β-Actin was used as a reference. *P<0.05, **P<0.01, ***P<0.001; two-tailed *t*-test.

To support our findings, cell migration and adhesion was studied in cSCC cells overexpressing PICSAR. First, cSCC cells were stably transfected with PICSAR expression vector and the level of overexpression was verified by qPCR (Fig. 3A). Levels of α2, α5 and β1 integrin mRNAs were significantly downregulated in stably PICSAR overexpressing cSCC cells (Fig. 3A). Also, expression of α2, α5 and β1 integrins on the cell surface, determined by flow cytometry, was decreased in PICSAR overexpressing cSCC cells (Fig. 3B).

The link between lncRNAs and integrin regulation is not well known and noted only in few studies (Zhang et al., 2014; Kim et al., 2013). Furthermore, the regulation of α2β1 or α5β1 integrins by lncRNAs has not been reported previously. Instead, microRNAs (miRNAs) have been shown to regulate expression of several integrin subunits, for example by targeting the 3′-UTR of integrin mRNAs (Chen et al., 2013). There are reports of lncRNAs targeting and inhibiting specific miRNAs but this competitive endogenous RNA (ceRNA) hypothesis has also been challenged (Thomson and Dinger, 2016). Here, using bioinformatics analysis, we did not find any potential miRNA-binding sites for PICSAR which could explain the regulation of integrin expression via altered miRNA expression after PICSAR knockdown or PICSAR overexpression in cSCC cells.

### Cell adhesion and spreading is decreased by PICSAR overexpression leading to increased migration of cSCC cells

To investigate the effect of PICSAR on cell spreading, PICSAR overexpressing cells were plated on collagen I and fibronectin. PICSAR overexpression resulted in impaired cell spreading and the relative number of spread cells was significantly decreased in PICSAR overexpressing cells compared to control cells (Fig. 3C). Immunofluorescence staining revealed expression of α2 and α5 integrins in PICSAR overexpressing cSCC cells plated on collagen I and fibronectin, but compared to the control cells there were no clear adhesion sites in PICSAR overexpressing cells, likely due to decreased cell spreading (Fig. 3D). In accordance with this, real-time cell adhesion assay revealed decreased adhesion of PICSAR overexpressing cSCC cells on collagen I and fibronectin, as compared to control cells (Fig. 3E). Furthermore, migration of PICSAR overexpressing cSCC cells in wound healing assay was increased compared to control cells (Fig. 3F). These results show for the first time a functional link between a lncRNA, PICSAR, and integrin regulation in cSCC cells leading to changes in the migratory phenotype of these keratinocyte-derived cancer cells.

The role of integrins in growth and differentiation of epidermal keratinocytes is well established, and mutations in integrin genes and changes in integrin expression have been documented in cSCC (Watt, 2002; Janes and Watt, 2006). In the epidermal layer of skin, α2 and β1 integrins are abundantly expressed by basal keratinocytes, which are in direct contact with intact basement membrane (Häkkinen et al., 1998), and their expression is downregulated during terminal differentiation of epidermal keratinocytes (Janes and Watt, 2006). Fibronectin receptor α5β1 is not expressed by keratinocytes in normal epidermis, but its expression is upregulated in hyperproliferative keratinocytes, for example in wound healing and in keratinocytes in culture (Larjava et al., 1993).

Activation of proto-oncogene Src has been linked to malignant transformation of tumor cells and in cSCC progression (Alt-Holland et al., 2011). Src also functions in other cellular processes such as cell adhesion, and it is an important regulator of integrin-mediated cell spreading and adhesion via direct interaction with integrin cytoplasmic domains transducing signals downstream of these cell surface receptors (Playford and Schaller, 2004). Moreover, Src plays an important role in cell motility by regulating focal adhesion turnover (Fincham and Frame, 1998). Introduction of viral Src (v-Src), a constitutively active variant of cellular Src (c-Src), into normal cells leads to disruption of cell-matrix adhesions and to cells adopting a rounded phenotype (Wang and Goldberg, 1976; Fincham et al., 1995). Both v-Src and c-Src are translocated by actin-dependent manner to focal adhesions where they induce activation of focal adhesion kinase (FAK) and disruption of cellular focal adhesions (Timpson et al., 2001; Fincham and Frame, 1998). In contrast, catalytically inactive c-Src-mutant suppresses FAK activation followed by increased actin stability and formation of enlarged focal adhesions, which eventually leads to decreased cell motility (Fincham and Frame, 1998). Here, decreased expression of Src in cSCC cells was noted after PICSAR knockdown (Fig. 3G; Fig. S4A,B). Moreover, expression of Src was markedly increased after PICSAR overexpression in cSCC cells (Fig. 3G; Fig. S4C), providing further evidence for a regulatory link between PICSAR and Src. These observations are consistent with previous studies, where Src has been shown to have an important role in cell adhesion and motility by regulating focal adhesion assembly and turnover (Fincham and Frame, 1998; Fincham et al., 1995; Wang and Goldberg, 1976; Timpson et al., 2001). Furthermore, the rate of cell migration correlates with the levels of integrin and ligand expression and changes in the affinity of integrin-ligand binding (Palecek et al., 1997). Cell adhesion increases when integrin expression increases, which contributes to a higher number of integrin–ligand bonds and a decreased migration rate (Palecek et al., 1997). Relatively small changes in integrin expression can lead to substantial changes in cell migration speed (Palecek et al., 1997). In addition, Src family kinases have been shown to regulate cell-matrix adhesion by modulating integrin affinity (Li et al., 2002). It is conceivable that optimal cell migration is dependent on the balance of integrin expression and Src activity for functional cell-matrix adhesion dynamics.

Progression of cSCCs is characterized by an alteration of basement membrane structure at an early stage, and by an eventual loss of basement membrane at invasive stage (Karppinen et al., 2016). There is also evidence that the cellular expression of lncRNAs is dependent on the composition and structure of the extracellular matrix (Zhuang et al., 2013; Li et al., 2017), indicating that the lncRNA expression varies depending on the activation of different adhesion receptors. We have previously noted that PICSAR is specifically expressed by tumor cells in cSCC, but not by keratinocytes in normal skin *in vivo* (Piipponen et al., 2016). It is therefore possible that during malignant transformation of epidermal keratinocytes, induction of PICSAR expression negatively regulates integrin expression, allowing detachment of cSCC cells from the basement membrane and invasion through an underlying dermal layer rich in collagen I.

The results of the present study show that PICSAR knockdown results in increased expression of α2β1 and α5β1 integrins on the cell surface, which explains the decreased migration of cSCC cells after PICSAR knockdown when cells adhere more efficiently on a collagen I and fibronectin coated surface. This hypothesis is further supported by experiments with PICSAR overexpressing cSCC cells, where we noted a decrease in integrin expression, resulting in decreased cell adhesion on collagen I and fibronectin, and increased cell migration. These results indicate a new mechanism for PICSAR in invasive cSCC by regulating cell migration by modifying the expression of collagen and fibronectin binding integrins.

## MATERIALS AND METHODS

### Cell cultures

Cutaneous SCC cell lines (UT-SCC12A and UT-SCC59) were established from surgically removed primary SCCs of the skin in Turku University Hospital (Riihilä et al., 2015) and cultured as previously described (Riihilä et al., 2015; Farshchian et al., 2015). The use of tumor samples was approved by the Ethics Committee of the Hospital District of Southwest Finland. All participants gave their written informed consent and the study was carried out with the permission of Turku University Hospital, according to the Declaration of Helsinki. The authenticity of all cSCC cell lines has been verified by short tandem repeat profiling (Farshchian et al., 2016).

### siRNA gene knockdown

cSCC cells were cultured to 50% confluence and transfected with negative control siRNA (AllStars Negative Control siRNA, Qiagen) and following commercially available siRNAs (75 nM, Qiagen) targeting two distinct areas in PICSAR 3′ end, as previously described (Piipponen et al., 2016): Hs_C21orf113_1, 5′-CACGGCCAACGTGGAGCTCTA-3′ (siRNA1); Hs_C21orf113_3, 5′-CTGCAGTCACTTCACAGTGAA-3′ (siRNA2).

### Recombinant PICSAR expression in cSCC cells

Specific primers for PICSAR variant expressed in cSCC cells were designed using Primer-BLAST (Ye et al., 2012) and purchased from Oligomer (Metabion International AG, Steinkirchen, Germany): forward primer 5′-CTGGCTCACCCTGGCACTG-3′, reverse primer 5′-CACCTAAGCAATGCAGAGAGG-3′. This fragment was amplified by PCR with Q5^®^ High-Fidelity DNA Polymerase (New England Biolabs, Ipswich, USA) using cSCC cell derived cDNA as template. PCR reaction was cycled at 98°C for 30 s and 40 cycles of amplification (98°C 10 s, 66°C 30 s and 72°C 30 s) followed by extension at 72°C for 10 min. The amplified DNA fragment was cloned into pcDNA3.1(-) (Invitrogen) containing the neomycin resistance gene. The construct was further sequenced to verify the orientation and integrity of the ligated *PICSAR* insert. cSCC cells were transfected with PICSAR expression construct (pcDNA3.1_PICSAR) or empty vector (pcDNA3.1) using Lipofectamine 3000 transfection reagent (Invitrogen) and transfected cells were selected with 1 µg/ml Geneticin (G418 sulfate, Invitrogen). Selective pressure of PICSAR overexpressing and control cell pools was maintained by using 500 µg/ml Geneticin.

### Cell migration assays

An ImageLock™ 96-well plate (Essen BioScience, Ann Arbor, USA) was coated with fibronectin (Merck Millipore, Darmstadt, Germany) or collagen I (Advanced BioMatrix, San Diego, USA) (both 5 µg/cm^2^). PICSAR siRNA and control siRNA treated cSCC cells were plated 72 h after siRNA transfection and cells were imaged using the IncuCyte ZOOM^®^ real-time cell imaging system (Essen BioScience). Individual migrating cells (*n*=15) were tracked (t=20 h) using the ImageJ software (Schneider et al., 2012). To examine cell migration in a wound healing assay, PICSAR expression construct (pcDNA3.1_PICSAR) and control expression vector (pcDNA3.1) transfected cSCC cells were plated on an ImageLock™ 96-well plate and incubated for 6 h with 1 mM hydroxyurea (Sigma-Aldrich) to prevent cell proliferation. Cell monolayer was scratched with IncuCyte wound-maker (Essen BioScience) simultaneously in all wells and incubation was continued in 1% fetal calf serum (FCS) containing growth media with 0.5 mM hydroxyurea. Cells were imaged every 10 min using the IncuCyte ZOOM^®^ and the results were analyzed with the IncuCyte ZOOM 2014A software (Essen BioScience).

### Cell adhesion and spreading assay

To study dynamic cell adhesion PICSAR siRNA and control siRNA treated cSCC cells (72 h after transfection), or PICSAR expression construct (pcDNA3.1_PICSAR) and control expression vector (pcDNA3.1) transfected cSCC cells were seeded on a collagen I or fibronectin coated (5 µg/cm^2^) E-Plate 96 (Roche Applied Sciences) and cell adhesion was measured using the xCELLigence real-time cell analyzer (RTCA; Roche Applied Sciences). The cell index indicates the readout of the microelectrode impedance, which corresponds to the strength of cell adhesion. The formation of lamellipodia was studied by microscopic quantitation in PICSAR siRNA and control siRNA treated cSCC cells 72 h after transfection. Cell spreading was studied in stably PICSAR overexpressing cSCC cells by microscopic quantitation. Cells were plated on collagen I or fibronectin coated 96-wells, and fixed 4 h after plating [8% formaldehyde, 10% sucrose in phosphate buffer saline (PBS) supplemented with 1 mM MgSO_4_ and 1 mM CaCl_2_]. The number of cells with lamellipodia or the number of spread cells was compared to the total cell number in each well (*n*=3) with a 20x objective.

### Visualization of RNA sequencing and quantitative real-time PCR

Morpheus software was used for gene expression visualization and to generate heatmaps (https://software.broadinstitute.org/morpheus) from RNA-seq data previously published by us (GEO accession number GSE77950) (Piipponen et al., 2016). Primers and probes for PICSAR and α2, α5 and β1 integrins were designed as previously described (Piipponen et al., 2016; Mirtti et al., 2006). The sequences for primers and probes are listed in the supplementary material online (Table S1). All qPCR reactions were performed using the QuantStudio 12K Flex (Thermo Fisher Scientific) system at the Finnish Functional Genomics Centre (FFGC) in Turku, Finland. Samples were analyzed using the standard curve method in three parallel reactions with threshold cycle (Ct) values <5% of the mean Ct. β-Actin mRNA was used as reference.

### Flow cytometry

PICSAR siRNA and control siRNA treated cSCC cells (72 h after transfection), or PICSAR expression construct (pcDNA3.1_PICSAR) and control expression vector (pcDNA3.1) transfected cSCC cells were analyzed for the levels of cell surface integrins α2, α5 and β1. Cells were detached from plates by using 0.25% Trypsin-Ethylenediaminetetraacetic acid (EDTA) and incubating in +37° for 5–10 min. Detached cells, 700,000 cells per sample, were suspended in blocking buffer in 1% FCS-PBS and incubated on ice for 30 min. No fixation or permeabilization was done to the cells to prevent intracellular antigen staining. After blocking, cells were stained with monoclonal antibodies (all in 1:100 dilution) specific for integrin α2 (555668), integrin α5 (555615) and integrin β1 (553715) (all from BD Biosciences) in 1% FCS-PBS for 1 h in +4°C. Cells were washed with PBS and highly precross-absorbed Alexa Fluor^®^ 488 conjugated goat anti-mouse antibody (Invitrogen) was used as secondary antibody in 1:200 dilution and cells were analyzed using FACSCalibur flow cytometer (BD Biosciences). The results were analyzed with Flowing Software 2.5.1 (Turku Centre for Biotechnology, University of Turku, Finland, www.flowingsoftware.com).

### Immunofluorescence assays

PICSAR siRNA and control siRNA treated cSCC cells (72 h after transfection), or PICSAR expression construct (pcDNA3.1_PICSAR) and control expression vector (pcDNA3.1) transfected cSCC cells were plated on cell culture plates coated with collagen I or fibronectin. Cells were fixed 4 h after plating with 4% paraformaldehyde-PBS, blocked with 3% bovine serum albumin (BSA) containing PBS and permeabilized with 0.2% Triton X-100-PBS. Cells were labeled with primary antibodies against integrin α2 (MCA2025, Serotec, Raleigh, USA) or integrin α5 (AB1949, Merck Millipore) in 1:100 dilution in blocking solution. Alexa Fluor^®^ 568 conjugated goat anti-mouse or anti-rabbit antibody (Invitrogen) was used as secondary antibody in 1:200 dilution together with Alexa Fluor^®^ 488 conjugated phalloidin in 1:50 dilution (Invitrogen). Hoechst (Invitrogen) was used to visualize nuclei. Cells were mounted on culture plates with Mowiol-DABCO (Sigma-Aldrich) and samples were examined with Zeiss AxioVert 200 M (Carl Zeiss, Jena, Germany) fluorescence microscope.

### Western blot analysis

Cell lysates of PICSAR and control siRNA transfected cultures, and PICSAR overexpressing and pcDNA3.1 vector containing cSCC cells were analyzed with antibodies specific for phosphorylated Src (pSrc, 2101) and total Src (2108, both from Cell Signaling Technology) in 1:1000 dilution. β-Actin (A1978, Sigma-Aldrich) expression level was used as loading control. Protein expression was quantitated using fluorescently labeled secondary antibodies in 1:15000 dilution (LI-COR Biosciences, Lincoln, NE) and the LI-COR Odyssey^®^ CLx fluorescent imaging system.

### Bioinformatic analysis of miRNA binding sites

To analyze potential miRNA target sites in PICSAR, following computational programs with different algorithms for miRNA-binding prediction were used; miRWalk (http://mirwalk.umm.uni-heidelberg.de/) (Dweep and Gretz, 2015), TargetScan (http://www.targetscan.org) (Agarwal et al., 2015) and DIANA–lncBase v2 (www.microrna.gr/LncBase) (Paraskevopoulou et al., 2015).

### Statistical analysis

Statistical analysis was performed using SPSS Statistics for Windows, v. 20.0. (IBM, Armonk, USA). Unpaired two-tailed *t*-test and Mann–Whitney two-way *U*-test were used. Statistical analysis for RNA-seq analysis was performed using the Limma package (Piipponen et al., 2016).

## Supplementary Material

Supplementary information
