# Supplementary material for: Long non-coding RNA PICSAR decreases adhesion and promotes migration of squamous carcinoma cells by downregulating α2β1 and α5β1 integrin expression
Source: Biol Open. 2018 Nov 15;7(11):bio037044. doi: 10.1242/bio.037044 (PMC6262852; doi:10.1242/bio.037044)
Supplement: Supplementary information [file biolopen-7-037044-s1.pdf]

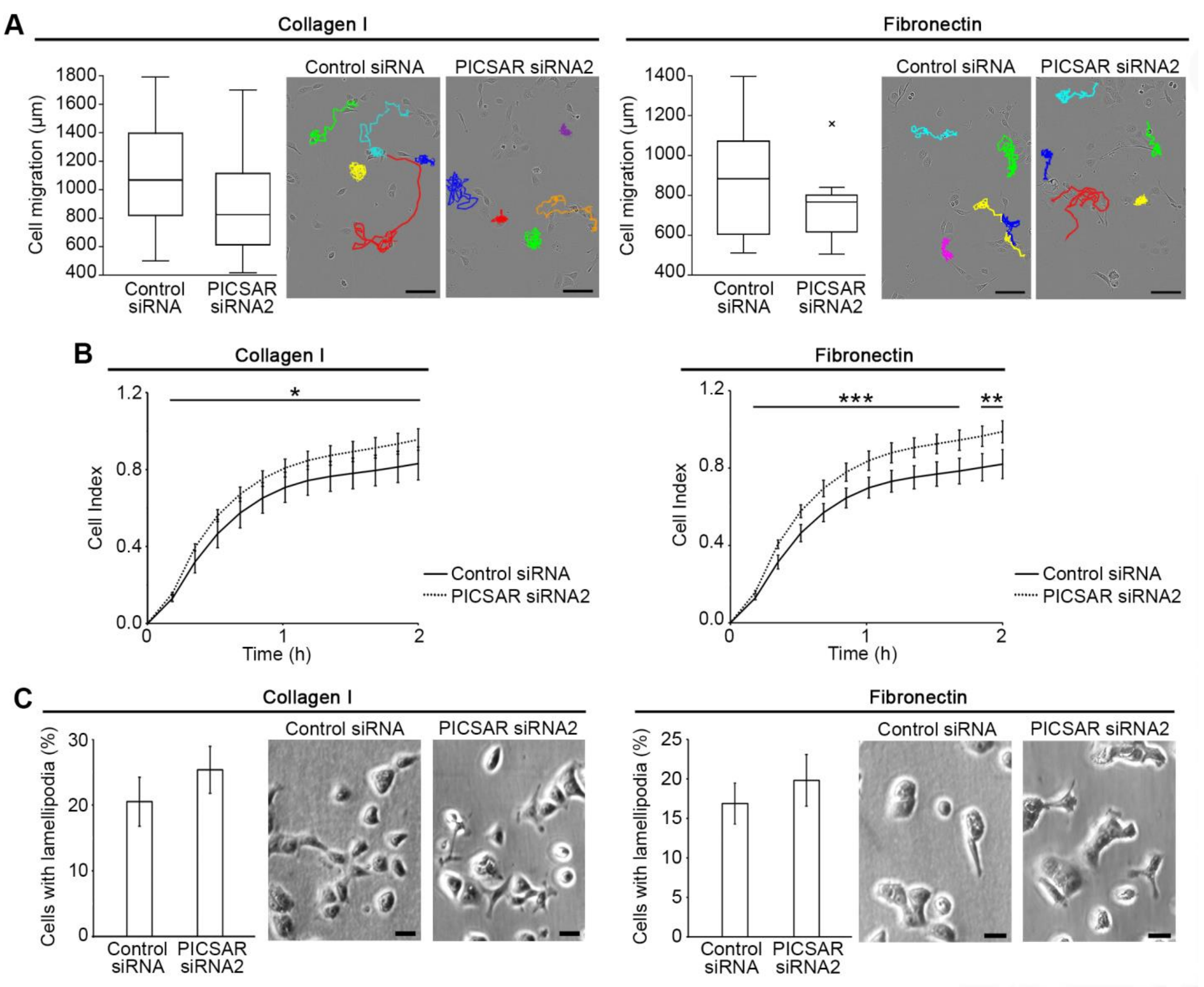

Fig. S1. Knockdown of PICSAR inhibits cSCC cell migration and increases cSCC cell adhesion and formation of lamellipodia. cSCC cells (UT-SCC12A) were transfected with PICSAR siRNA (siRNA2) or control siRNA. (A) cSCC cells were plated on collagen I or fibronectin 72 hours after transfection and migration of individual cells was imaged using the IncuCyte ZOOM® real-time cell imaging system. Cell tracking (n=15) was quantitated using ImageJ software. Median±s.d. is shown. Representative images of cell tracking are shown. Scale bar=100 µm. (B) cSCC cells were plated on collagen I or fibronectin coated 96-well electronic microtiter plate 72 hours after transfection and cell adhesion was measured using the xCELLigence system (n=3). Mean±s.d. is shown; *P<0.05, **P<0.01, ***P<0.001; two-tailed t-test. (C) cSCC were plated on collagen I or fibronectin 72 hours after transfection and fixed 4 hours after plating. The number of lamellipodia containing cells was quantitated from microscopic images at 20x magnification (n=3). Representative images of the quantitation are shown. Scale bar=10 µm. Mean±s.d. is shown.

Biology Open • Supplementary information

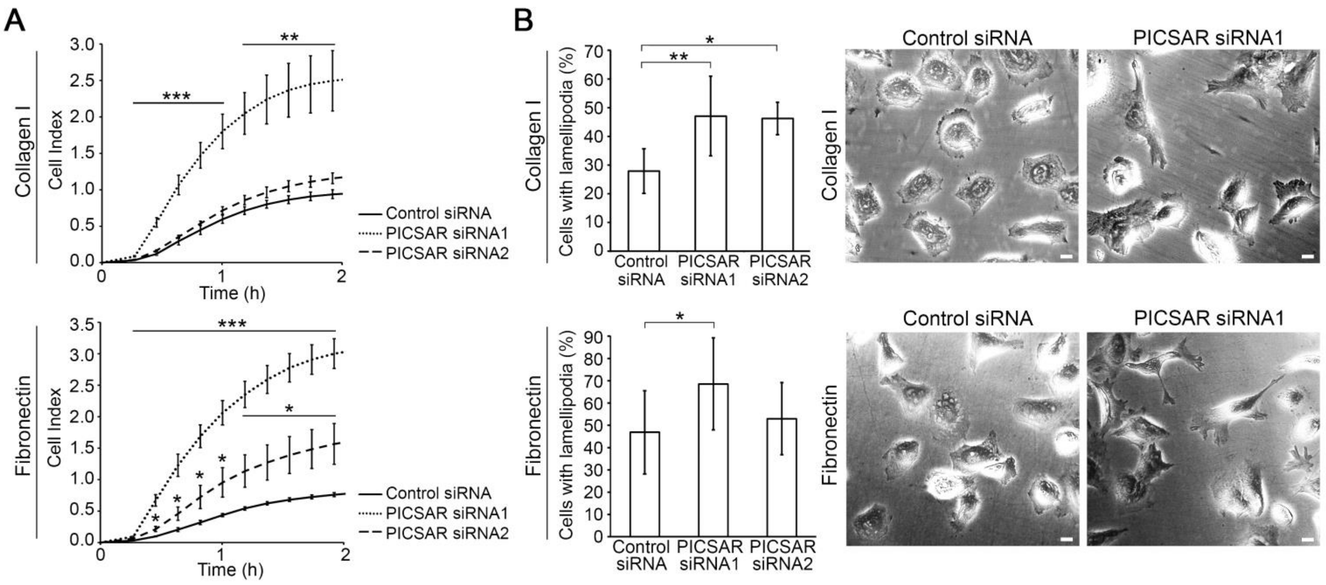

Fig. S2. Knockdown of PICSAR increases cSCC cell adhesion and formation of lamellipodia. cSCC cells (UT-SCC59A) were transfected with PICSAR siRNAs (siRNA1 or siRNA2) or control siRNA. (A) Cells were plated on collagen I or fibronectin coated 96-well electronic microtiter plate 72 hours after transfection and cell adhesion was measured using the xCELLigence system (n=3). Mean±s.d. is shown. (B) After 72 hours of transfection cells were plated on collagen I or fibronectin coated wells. Cells were fixed 4 hours after plating and the number of lamellipodia containing cells was quantitated from microscopic images (n=3) at 20x magnification (left panel). Mean±s.d. is shown. Representative images of the quantification are shown (right panel). Scale bar=10 µm. *P<0.05, **P<0.01, ***P<0.001; two-tailed t-test.

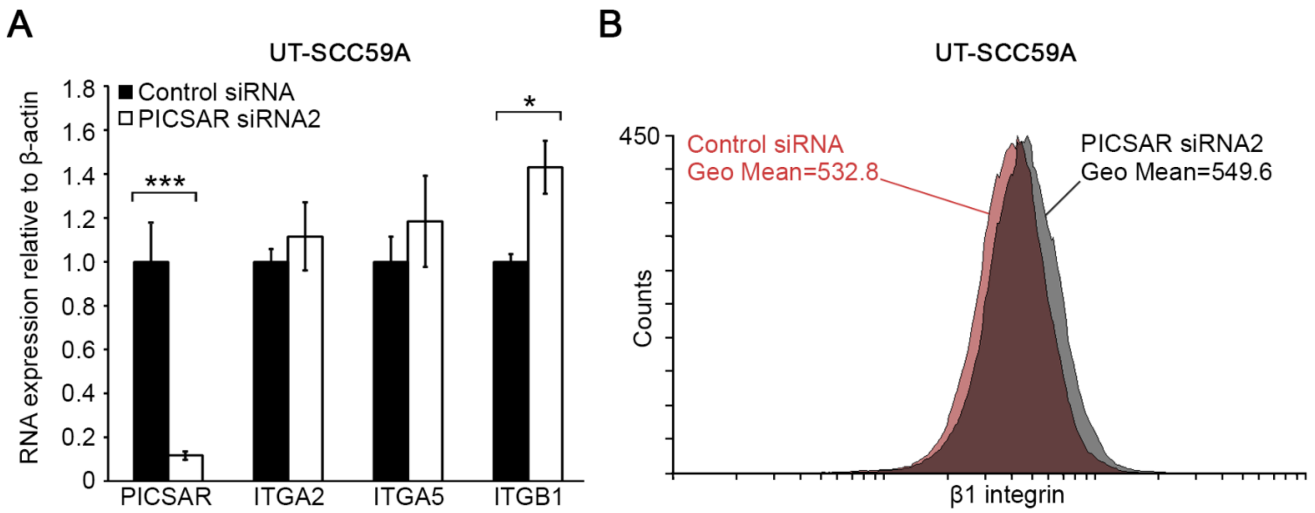

Fig. S3. Knockdown of PICSAR increases integrin expression in cSCC cells. cSCC cells (UT-SCC59A) were transfected with PICSAR siRNA (siRNA2) or control siRNA and incubated for 72 hours. (A) Levels of PICSAR and α2, α5 and β1 integrin mRNAs were determined with qPCR in PICSAR knockdown cells (n=3). Mean±s.d. is shown. *P<0.05, ***P<0.001; two-tailed *t*-test. (B) Flow cytometry was performed to measure β1 integrin expression on the cell surface in PICSAR knockdown cells.

Biology Open • Supplementary information

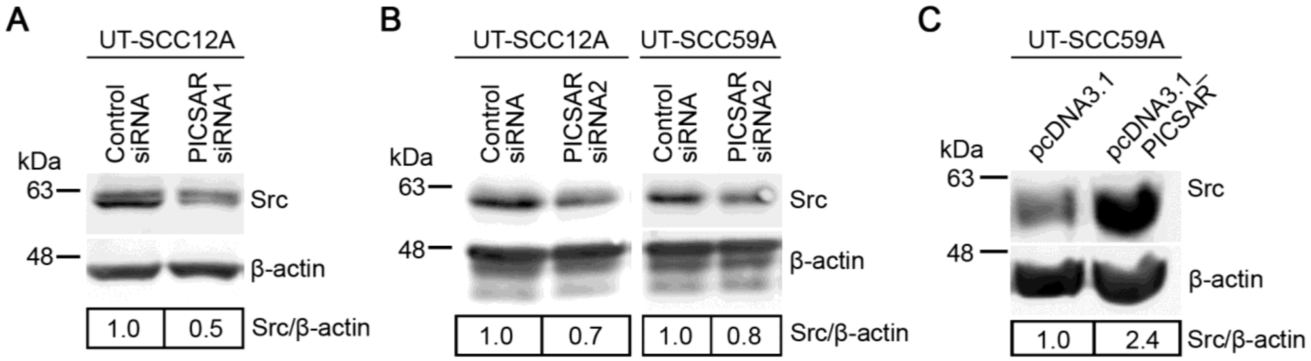

Fig. S4. Expression of Src is regulated by PICSAR in cSCC cells. (A) cSCC cells (UT-SCC12A) were transfected with PICSAR siRNA (siRNA1) or control siRNA. After 72 hours of transfection protein level for Src was determined by Western blotting analysis. (B) cSCC cells (UT-SCC12A and UT-SCC59A) were transfected with PICSAR siRNA (siRNA2) or control siRNA and protein level for Src was determined by Western blotting analysis 72 hours after transfection. (C) Protein level for Src was determined by Western blotting analysis of stably PICSAR overexpressing UT-SCC59A cells. The representative image and quantification shown are from one individual experiment. β-Actin was used as loading control.

Biology Open • Supplementary information

Table S1. List of specific primers and probes for qPCR.

Gene		Sequence
PICSAR	Forward	5′-TGCCTGGACTTTCAAGAGGTAA-3′
	Reverse	5′-GCTCTCAGTCAGCAGACACTT-3′
	Probe	5′-Fam-CCGAGCTCTGCTCTGAGGCCT-BHQ1-3′
ITGA2	Forward	5′-TGGATTTGCGTGTGGACATC-3′
	Reverse	5′-GGCAGTTCTAGAATAGGCTTCAA-3′
	Probe	5′-Fam-TCTGGAAAACCCTGGCACTAGCCCTG-BHQ1-3′
ITGA5	Forward	5′-GGGTGGCCTTCGGTTTACAG-3′
	Reverse	5′-GCTTTGCGAGTTGTTGAGATTC-3′
	Probe	5′-Fam-TCCCTCATCTCCGGGACACTAA-BHQ1-3′
ITGB1	Forward	5′-CAAGGGCAAACGTGTGAGA-3′
	Reverse	5′-TGAAGGCTCTGCACTGAACA-3′
	Probe	5′-Fam-TGTGTCAGACCTGCCTTGGTGTC-BHQ1-3′
ACTB	Forward	5′-TCACCCACACTGTGCCCATCTACGC-3′
	Reverse	5′-CAGCGGAACCGCTCATTGCCAATGG-3′
	Probe	5′-Fam-CAGCGGAACCGCTCATTGCCAATGG-BHQ1-3′

PICSAR, p38 inhibited cutaneous squamous cell carcinoma associated lincRNA; *ITGA2*, integrin α2; *ITGA5*, integrin α5; *ITGB1*, integrin ß1; *ACTB*, ß-Actin

Biology Open • Supplementary information
